# Socioeconomic inequalities in the use of outpatient services in Brazil according to health care need: evidence from the World Health Survey

**DOI:** 10.1186/1472-6963-10-217

**Published:** 2010-07-23

**Authors:** Célia L Szwarcwald, Paulo RB Souza-Júnior, Giseli N Damacena

**Affiliations:** 1 Fundação Oswaldo Cruz, Rio de Janeiro, Brasil

## Abstract

**Background:**

The Brazilian health system is founded on the principle of equity, meaning provision of equal care for equal needs. However, little is known about the impact of health policies in narrowing socioeconomic health inequalities. Using data from the Brazilian World Health Survey, this paper addresses socioeconomic inequalities in the use of outpatient services according to intensity of need.

**Methods:**

A three-stage cluster sampling was used to select 5000 adults (18 years and over). The non-response rate was 24.7% and calibration of the natural expansion factors was necessary to obtain the demographic structure of the Brazilian population. Utilization was established by use of outpatient services in the 12 months prior to the interview. Socioeconomic inequalities were analyzed by logistic regression models using years of schooling and private health insurance as independent variables, and controlling by age and sex. Effects of the socioeconomic variables on health services utilization were further analyzed according to self-rated health (good, fair and poor), considered as an indicator of intensity of health care need.

**Results:**

Among the 5000 respondents, 63.4% used an outpatient service in the year preceding the survey. The association of health services utilization and self-rated health was significant (p < 0.001). Regarding socioeconomic inequalities, the less educated used health services less frequently, despite presenting worse health conditions. Highly significant effects were found for both socioeconomic variables, years of schooling (p < 0.001) and private health insurance (p < 0.00), after controlling for age and sex. Stratifying by self-rated health, the effects of both socioeconomic variables were significant among those with good health status, but not statistically significant among those with poor self-rated health.

**Conclusions:**

The analysis showed that the social gradient in outpatient services utilization decreases as the need is more intense. Among individuals with good self-rated health, possible explanations for the inequality are the lower use of preventive services and unequal supply of health services among the socially disadvantaged groups, or excessive use of health services by the wealthy. On the other hand, our results indicate an adequate performance of the Brazilian health system in narrowing socioeconomic inequalities in health in the most serious situations of need.

## Background

The main purpose of health system performance assessment is to provide decision makers with reliable evidence for policy and decision making. It is an essential step to guarantee quality of care and to make decisions that satisfy the population needs and expectations [1].

The increasing evidence of the association between health and socioeconomic status from studies conducted throughout the world [2-4], using either individual characteristics such as education, income or ethnicity [5-8] or group characteristics to explain social and spatial variations in health [9,10], has promoted the monitoring of socioeconomic health inequalities as an important component of a health system performance assessment [11,12]. Actions and programs are evaluated based on their performance in diminishing the socioeconomic gradient [13,14], including the contribution of primary care [15,16].

In some countries, reducing inequalities in health has been identified as a key target in the context of government policies and strategic programs [17-21]. A research agenda to guide efforts for better understanding of interventions that promote equity has been proposed [15,22]. Over the last twenty years, this issue has been emphasized in the policy agenda of the World Health Organization (WHO), and has been considered as one of its first priorities [23-25].

The main argument for the reduction in health inequalities is based on the equity principle, which incorporates the dimension of social justice [26,27]. Nevertheless, even though the goal of health inequality reduction is founded on principles of social justice, a step forward is necessary to transpose these principles in concrete actions targeting inequality reduction. In practical terms, assessing health inequity within a society requires not only examining inequalities in health between more and less socially affluent groups, focusing on those inequalities likely to be avoidable [28], but also on specific actions known to effectively benefit the poorest [29].

Founded on the principle of equity, the Brazilian health system (SUS) provides universal access and comprehensive care, meaning equal provision of care for equal needs [30]. However, little is known about the impact of health policies in narrowing socioeconomic health inequalities.

As part of the World Health Organization (WHO) project focused on health systems performance assessment of the member countries [31], the World Health Survey (WHS) was carried out in Brazil in 2003. Using data from the Brazilian survey, this paper addresses the socioeconomic inequality in utilization of health services and its relationship with self-perception of health, as an indicator of need.

## Methods

The World Health Survey (WHS) was carried out in Brazil in 2003. The research was approved by the Research Ethics Committee of the Fundação Oswaldo Cruz - FIOCRUZ. Coordination of the survey and fieldwork as well as selection, training and supervision of interviewers were in charge of FIOCRUZ. The interviewers were undergraduate students or professionals from the health sector.

The survey population corresponded to the entire set of permanent private households in Brazil, except for those located in the rural areas of the Northern macro-region and special census tracts (military barracks and bases, lodgings, camps, ships/vessels, prisons, nursing homes, orphanages, convents/monasteries, and hospitals). According to this definition, the sample population included 207,513 tracts (96.2% of the 215,811 census tracts from 2000). According to the 2000 Population Census, of the 45,053,286 permanent private households existing in Brazil, 44,005,362 (97.7%) were covered by the sample population.

To ensure that the sample was representative of the urban and rural areas of the small, medium, and large municipalities, which have important differences in the size and type of health services, the tracts were divided into six strata based on urban/rural area and the municipality population size (< 50,000 inhabitants; 50,000-399,999; and 400,000 +).

A three-stage cluster sampling was used to select 5000 adults (18 years and over). In the first stage, 250 census tracts were systematically selected, with probability proportional to size. Within each stratum, the tracts were ordered (before their selection) according to mean income (implicit stratification), which guaranteed the representation of all the socioeconomic levels in each stratum. In the second stage, households were randomly selected using an inverse sample design to assure 20 interviews by sector [32]. In each household, one adult (18 years and over) was randomly selected to answer a face-to-face interview and one household member was identified to respond to questions relative to household characteristics, assets and expenditures. The random selection method proposed by the WHO consisted of using Kish numbers (numbers selected with equal probability from the sets of natural numbers with 2, 3, 4, 5, or 6 elements), which was adapted in the Brazilian survey for households with up to 12 adult residents [32].

In all 250 sample tracts, all 20 planned interviews were obtained, and an average of 34.4 households visits were made. The mean number of households visited (34.4) includes 20 permanent private households interviewed (58.1%); 8.5 refusals (24.7%); 3.3 permanent private households that were vacant (9.6%); and 2.6 non-existent households or dwelling units that were no longer permanent private households (7.6%).

Natural expansion factors in the design were based on the inverse probability of the selection of a household and the selection of an adult. Using the natural expansion factors, the age distribution showed the same pattern as the total population but an overestimation of the adult female population was found. Distribution by income quintiles showed a similar pattern to that described for the total population, despite of the slight underestimation in the wealthiest fifth. Further calibration of the natural expansion factors was necessary to obtain the census demographic structure of the Brazilian population [32].

The questionnaire originally proposed by the World Health Organization (WHO) was entirely reviewed and adapted to the Brazilian context. The following modules were included in the Brazilian WHS: socioeconomic status; self-rated health; risk factors; chronic diseases; coverage of health programs; responsiveness; and health expenditures, including private health insurance, health care services and products, diagnostics and laboratory tests, and medications.

For the statistical analysis, the sampling design was taken into account and all statistical analyses were conducted using SUDAAN [33].

Health services utilization was established by use of outpatient health care services in the 12 months prior to the interview.

Analysis of health status was based on the following question: *"In general, how would you rate your health today"*? Responses varied on a scale of 1 to 5 (1 = very bad; 2 = bad; 3 = fair; 4 = good; 5 = very good). The responses were aggregated to establish "good self-rated health" (good or very good), "fair" and "poor self-rated health (bad or very bad), which was used as an indicator of intensity of health care need.

To examine inequalities by socioeconomic status, two variables were considered: years of schooling and having private health insurance. Utilization of health services and self-rated health were jointly analyzed by educational level.

The χ^2 ^statistical test was used to analyze the association between health services utilization and self-rated health.

The effects of the socioeconomic factors on health care utilization were analyzed by logistic regression models using years of schooling and private health insurance as independent variables, after controlling for age and sex.

Further multivariate analyses were conducted, considering self-rated health as an indicator of intensity of health care need. After controlling by age, effects of the socioeconomic variables (years of schooling and private health insurance) on health services utilization were analyzed according to health status (good, fair and poor) and sex.

## Results

Of the 5000 respondents, 63.4% used an outpatient service in the year preceding the survey. As shown in Table 1, higher percentages were found among women (69.0%) when compared to men (56.9%). The proportion of utilization varied with perception of health status, from 59.2%, among those with good self-rated health, to 71.6%, among those with poor health status. The association of outpatient services utilization and self-rated health was significant only for those of lower socioeconomic status, indicated by either one of the two variables. Overall, 28.4% with poor health status did not use outpatient services in the previous year, 25.9% among women and 32.4% among men, and had their needs unmet.

**Table 1:** Percentage of outpatient services utilization in the previous year according to self-rated health and educational level and private health insurance

Self-Rated Health	Educational level				Total	
			
	Incomplete elementary school		Complete elementary school and over			
	
	n	%	n	%	n	%
**Good/Very good**	1135	51.8	1528	64.6	2663	59.2

**Fair**	1257	66.2	615	70.2	1872	67.5

**Bad/Very bad**	379	72.1	83	69.1	462	71.6

**Total**	2771	61.1	2226	66.3	4997	63.4

p-value*	<0.001		0.083		<0.001	

**Self-Rated Health**	**Private health insurance**				**Total**	
			
	**No**		**Yes**			
	
	**n**	**%**	**n**	**%**	**n**	**%**

**Good/Very good**	1804	52.0	842	74.2	2647	59.1

**Fair**	1483	65.7	381	74.4	1863	67.5

**Bad/Very bad**	398	71.0	63	76.1	461	71.7

**Total**	3685	59.6	1286	74.3	4971	63.4

p-value*	<0.001		0.952		<0.001	

*Significance of the χ^2 ^test of differences in proportions across categories of self-rated health status.

**Figure 1:**
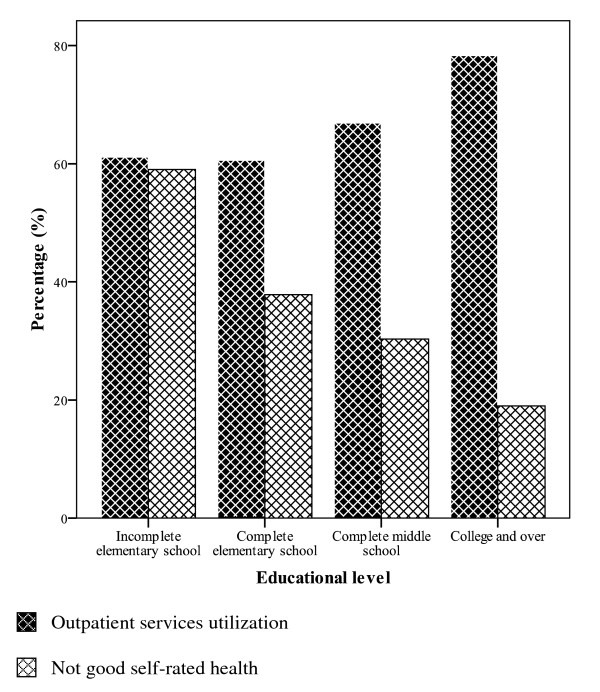
Percentage of individuals with not good self-rated health (fair or poor) and percentage of outpatient services utilization in the previous year by educational level

Figure 1 shows the proportions of health services utilization in the previous year and poor self-rated health by educational level. The social gradients are in opposite directions: although the less educated have the greatest proportion of poor self-rated health, they are the ones with smaller health care utilization.

The results of logistic regression presented in Table 2 show the effects of age and sex on outpatients services utilization in the previous year: higher rates are found among women when compared to men (p < 0.001), and among the elderly when compared to the youngest (p = 0.039). Moreover, a highly significant effect was found for years of schooling (p < 0.001), indicating a more frequent utilization of health services among more educated people, after controlling for the effects of age and sex. As to health insurance, the odds of utilization is almost two times greater (OR = 1.964) among those holding a private health plan.

**Table 2:** Results of multivariate logistic analyses: effects of age, sex and socioeconomic variables on outpatient services utilization

Model I			
**Independent variables**	Exp(b)*	**CI(95%)**	**p-value**

Age	1.004	(1.000 - 1.008)	0.039

Sex	0.595	(0.519 - 0.682)	<0.001

Constant	3.139	(2.396 - 4.111)	<0.001

**Model II**			

**Independent variables**	Exp(b)*	**CI(95%)**	**p-value**

Age	1.011	(1.006 - 1.015)	<0.001

Sex	0.589	(0.513 - 0.676)	<0.001

Years of schooling	1.056	(1.038 - 1.074)	<0.001

Constant	1.730	(1.235 - 2.424)	0.002

**Model III**			

**Independent variables**	Exp(b)*	**CI(95%)**	**p-value**

Age	1.003	(0.999 - 1.007)	0.154

Sex	0.585	(0.508 - 0.675)	<0.001

Private health insurance	1.964	(1.653 - 2.333)	<0.001

Constant	2.881	(2.190 - 3.789)	<0.001

*Exponential of logistic regression estimated parameter.

Models using health services utilization as the dependent variable and stratified by self-rated health (good, fair and poor) are shown in Table 3. Different effects of the independent variables on the utilization of outpatient services (after controlling by age and sex) are observed by strata. Among those with good health state perception, the effect of years of schooling was highly significant (OR = 1.086; p < 0.001). Among those with fair evaluation of health state, the social gradient decreases yet statistically significant (OR = 1.050; p = 0.002). However, for those with poor health status, the effect of educational level disappears (OR = 0.992).

**Table 3:** Results of multivariate logistic analyses stratified by self-rated health status: effects of years of schooling on outpatient services utilization, after controlling for age and sex

**Good self-rated health **(n = 2639)			
**Independent variables**	Exp(b)*	**CI(95%)**	**p-value**

Age	1.007	(1.000-1.013)	0.042

Sex	0.611	(0.507-0.737)	<0.001

Years of schooling	1.086	(1.064-1.109)	<0.001

Constant	1.256	(0.805-1.960)	0.314

**Fair self-rated health **(n = 1849)			

**Independent variables**	Exp(b)*	**CI(95%)**	**p-value**

Age	1.008	(1.001-1.015)	0.025

Sex	0.615	(0.492-0.768)	<0.001

Years of schooling	1.050	(1.018-1.083)	0.002

Constant	2.222	(1.321-3.737)	0.003

**Poor self-rated health **(n = 459)			

**Independent variables**	Exp(b)*	**CI(95%)**	**p-value**

Age	0.996	(0.982-1.012)	0.646

Sex	0.720	(0.438-1.185)	0.195

Years of schooling	0.992	(0.925-1.064)	0.822

Constant	4.878	(1.741-13.670)	0.003

*Exponential of logistic regression estimated parameter.

Similar results were obtained using private health insurance as the independent variable (Table 4). The odds ratio decreases from 2.672 among those that reported good health to 1.290 among individuals with poor self-rated health and the effect was not statistically significant.

**Table 4:** Results of multivariate logistic analyses stratified by self-rated health status: effects of private health insurance on outpatient services utilization after controlling for age and sex

Good self-rated health (n = 2647)			
**Independent variables**	Exp(b)*	**CI(95%)**	**p-value**

Age	0.995	(0.989-1.001)	0.112

Sex	0.604	(0.499-0.730)	<0.001

Private health insurance	2.672	(2.129-3.353)	<0.001

Constant	2.803	(1.929-4.073)	<0.001

**Fair self-rated health **(n = 1863)			

**Independent variables**	Exp(b)*	**CI(95%)**	**p-value**

Age	1.002	(0.995-1.008)	0.588

Sex	0.619	(0.494-0.775)	<0.001

Private health insurance	1.521	(1.129-2.050)	0.006

Constant	3.484	(2.252-5.392)	<0.001

**Poor self-rated health **(n = 461)			

**Independent variables**	Exp(b)*	**CI(95%)**	**p-value**

Age	0.997	(0.985-1.010)	0.662

Sex	0.729	(0.442-1.203)	0.215

Private health insurance	1.290	(0.645-2.582)	0.470

Constant	4.416	(2.034-9.588)	<0.001

*Exponential of logistic regression estimated parameter.

## Discussion

Although health inequalities have most often been documented in wealthy countries, recent studies have made it evident that inequality in health and health care is also a prominent problem in developing countries [28,34-36]. In particular, analysis of the effect of health care is especially important in those countries, where resource limitations require that the effective use of all health interventions be made.

The results of the present study show the existence of significant socioeconomic inequalities in health services utilization. The rate of outpatient services utilization is higher among individuals who have private health insurance and higher level of education. Furthermore, the analysis shows social gradients in opposite directions: the less educated have the largest proportion of poor self-rated health but are the ones with lower utilization rate. Following the inverse care law [37], use of care varies inversely with the needs of the population served.

In Brazil, the association between utilization rate and socioeconomic status has been shown before [38,39], and is partly explained by the influence of private health plan coverage, since persons with private health insurance have shown higher odds of utilizing health care services [40]. Another factor is the limited capacity of public outpatient services to meet the demand of health care [41-43].

In relation to differences in health service utilization by sex, higher percentages of service utilization were found among women. Gender differences in health care utilization are well-known. In general, studies that address morbidity and health services utilization have found that women report more symptoms than men, have worse perception of health status, and are more likely to use medical care [44].

Our findings indicate a consistent association between the use of outpatient services and health care need, as individuals reporting poor health were more likely to use health care, corroborating results from other studies [45-47]. Yet, it is important to note that more than a quarter did not use an outpatient service in the preceding year despite reporting a fair or poor self-rated health, or having unmet health care needs. This issue deserves specific attention as studies have related unmet health care needs with an increase in mortality risk [48].

In regard to the association between socioeconomic status and health services utilization, according to self-rated health status, the analysis shows that the weaker the need, the sharper the socioeconomic gradient in health services utilization. The effects of both variables (private health insurance and years of schooling) are pronounced in the group with good self-rated health but decrease and are not significant among those with poor health status. While the wealthiest have high utilization rates regardless of self-perception of health problems, individuals of low socioeconomic status have to feel ill to seek health care.

In the group with good self-rated health, a possible explanation of the increased use among the richest is the higher use of preventive services. Differences in the main reason for seeking health care among rural and urban populations were evidenced in a previous study in Brazil: while use of preventive services was predominant in urban areas, presence of disease was the main reason in the rural population, which has lower socioeconomic status and more difficulties in the access of health services [49].

Unequal access to the services provided is another possible explanatory mechanism. Even though universal access to health services is guaranteed by the Brazilian constitution, which has allowed for improvements of many aspects in health [50,51], some studies have shown inequality in the geographic distribution of available resources, mainly those requiring a more sophisticated technology for diagnosis [52,53].

The availability, the type and the quantity of services and resources (financial, human, and technological) are aspects of supply that may be influencing the pattern of utilization of health services in Brazil. A study using data from the National Household Sample Survey (2003) evidenced lower utilization by elderly rural residents when compared to old residents of urban areas, even among those who reported health problems. Furthermore, analysis showed that there was limited access to services with intermediate complexity [54].

An alternative explanation of the pro-rich inequality in health services utilization among those with good health status is that the wealthiest are more likely to use health services excessively, mainly among those that have a private health care plan. Our results show that the odds of outpatient services utilization is 2.7 greater among those with private health insurance than among those without private insurance. As has been pointed out by Starfield and collaborators [55], the excessive use of health services is becoming increasingly evident and deserves attention, as it is associated with higher costs, more medical procedures and more medications, without producing differences in quality of services [56]. In Brazil, excessive medical interventions during pregnancy and childbirth in the private sector may be influencing the increase in preterm deliveries, diminishing the gains resulting from improved antenatal care and increased newborn survival [57].

However, neither having private health insurance nor the availability of health services can ensure utilization of health services. Social, cultural and environment aspects are factors that could possibly influence health care use [58]. In Brazil, the lack of knowledge about disease prevention must also be considered, which particularly affects healthy habits, lifestyles and utilization of health care services [59,60].

On the other hand, the analysis evidenced that the effects of socioeconomic variables on health services utilization are reduced as the level of self-rated health status worsens, or as the health care need is more intense. Furthermore, among those with poor self-rated health, socioeconomic differences in health services utilization were not significant.

So, as compared to previous studies on inequalities in health care utilization [38-43], the present study shows encouraging news. The findings indicate that access and utilization of health services are provided to the population with perceived health problems regardless of educational level and are particularly relevant in the context of reducing socioeconomic health inequalities. Clearly, the evidence depicted here deserves further analysis, taking into account indicators of quality of care received.

Another limitation of this survey is that our measure of health services utilization is based on self-reported data. It is well known that such data are subject to measurement error that arises when respondents are asked to recall past utilization. So, the results here presented should be interpreted in the light of this limitation [61].

## Conclusions

Our findings are important to the evaluation of the Brazilian health system performance. The evidence shows that the less educated use less frequently outpatient services, despite presenting worse health conditions, that is, health service utilization varies inversely with the needs of the population served.

However, socioeconomic inequalities in health services utilization decrease and even disappear in the most serious situations of need. Therefore, our results indicate a good performance of the Brazilian health system in narrowing socioeconomic inequalities in health, especially when the need is more intense. On the other hand, our results indicate the need to develop health promotion policies and to expand the health services supply in accordance with the territorial, cultural and social characteristics of the Brazilian population. Excessive use of medical services in the private health sector is another aspect that might be influencing the pattern of health services utilization and deserves further investigation.

## Competing interests

The authors declare that they have no competing interests.

## Authors' contributions

CLS conceived and designed the article content, participated in the data analysis and drafting of the article, and coordinated the survey. PRBSJ was responsible for the data analysis and supervised the survey in some regions. GND participated in drafting the article and in the literature review. All authors read and approved the final manuscript.

## Pre-publication history

The pre-publication history for this paper can be accessed here:

http://www.biomedcentral.com/1472-6963/10/217/prepub
